# Multiple Myeloma Bone Disease: Implication of MicroRNAs in Its Molecular Background

**DOI:** 10.3390/ijms22052375

**Published:** 2021-02-27

**Authors:** Aristea-Maria Papanota, Paraskevi Karousi, Christos K. Kontos, Ioannis Ntanasis-Stathopoulos, Andreas Scorilas, Evangelos Terpos

**Affiliations:** 1 Department of Clinical Therapeutics, School of Medicine, National and Kapodistrian University of Athens, 11528 Athens, Greece; ampapanota@med.uoa.gr (A.-M.P.); johnntanasis@med.uoa.gr (I.N.-S.); 2 Department of Biochemistry and Molecular Biology, Faculty of Biology, National and Kapodistrian University of Athens, 15701 Athens, Greece; pkarousi@biol.uoa.gr (P.K.) ; chkontos@biol.uoa.gr (C.K.K.); ascorilas@biol.uoa.gr (A.S.)

**Keywords:** miRNAs, small non-coding RNAs, RANK/RANKL pathway, WNT pathway, SMAD, NOTCH pathway, extracellular vesicles, regulators, molecular biomarkers, MMBD

## Abstract

Multiple myeloma (MM) is a common hematological malignancy arising from terminally differentiated plasma cells. In the majority of cases, symptomatic disease is characterized by the presence of bone disease. Multiple myeloma bone disease (MMBD) is a result of an imbalance in the bone-remodeling process that leads to increased osteoclast activity and decreased osteoblast activity. The molecular background of MMBD appears intriguingly complex, as several signaling pathways and cell-to-cell interactions are implicated in the pathophysiology of MMBD. MicroRNAs (miRNAs) are small non-coding RNA molecules that regulate the expression of their target mRNAs. Numerous miRNAs have been witnessed to be involved in cancer and hematological malignancies and their role has been characterized either as oncogenic or oncosuppressive. Recently, scientific research turned towards miRNAs as regulators of MMBD. Scientific data support that miRNAs finely regulate the majority of the signaling pathways implicated in MMBD. In this review, we provide concise information regarding the molecular pathways with a significant role in MMBD and the miRNAs implicated in their regulation. Moreover, we discuss their utility as molecular biomarkers and highlight the putative usage of miRNAs as novel molecular targets for targeted therapy in MMBD.

## 1. Introduction

**Figure 1:**
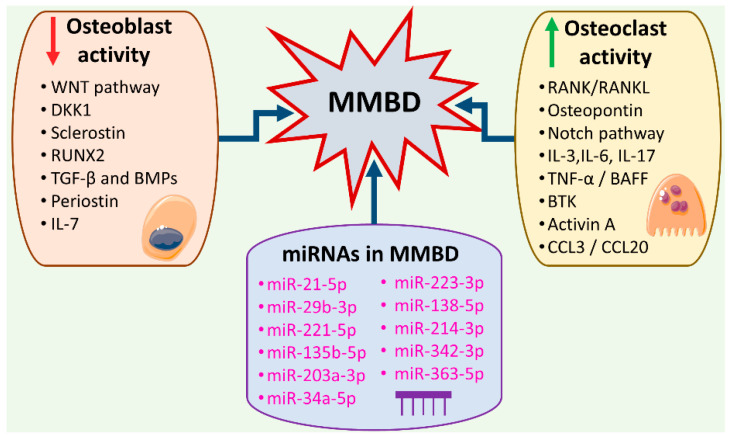
A brief description of the molecular background of multiple myeloma bone disease (MMBD), showing molecules and pathways affecting osteoblast and osteoclast activity, as well as the main microRNAs (miRNAs) playing a role in MMBD.

Multiple myeloma (MM) is a malignancy arising from terminally differentiated plasma cells that reside in the bone marrow and represents the second most common hematological cancer following non-Hodgkin lymphomas [1]. In the majority of cases, the symptomatic disease is characterized by the presence of end-organ damage as summarized by the acronym CRAB, where “B” stands for bone disease [2]. Whole-body low-dose computed tomography (WBLDCT) is currently considered the standard imaging method for detecting MM bone disease (MMBD) [3,4]. MMBD arises from an imbalance in normal bone remodeling caused by increased osteoclast and decreased osteoblast activity [5]. The bone marrow microenvironment consists of mineralized extracellular matrix and cellular components which are besides osteocytes, osteoclasts, and osteoblasts also stromal cells, and immune cells. In MMBD, molecules produced by the cellular components of the bone marrow microenvironment trigger an imbalance between bone destruction and bone formation. The molecular background of MMBD appears intriguingly complex, as several signaling pathways and cell-to-cell interactions are implicated in the pathophysiology of MMBD [6,7,8]. A brief description of the molecular background of MMBD is illustrated in Figure 1. About 70–80% of newly diagnosed MM patients present with bone involvement in the form of osteolysis and are at increased risk of presenting skeletal-related events (SREs). SREs are namely: spinal cord compression, pathologic fracture, need for palliative radiotherapy or need for surgical intervention. SREs are responsible for the poor quality of life and worse survival of MM patients [9,10]. Regarding the management of MMBD, the approach is multidisciplinary, ranging from bisphosphonates and targeted MM therapies (i.e., denosumab) to radiation and orthopedic surgery [11]. In today’s clinical practice bisphosphonate treatment is indicated in all newly diagnosed MM patients receiving frontline treatment regardless of the presence of bone lesions in conventional radiography. Among bisphosphonates, zoledronic acid is preferred, since it is associated with improved progression-free (PFS) and overall survival (OS) in patients with newly diagnosed MM and MMBD, while denosumab is suitable for all patients with MMBD, especially in those with renal impairment [11]. Furthermore, denosumab seems to increase PFS in subpopulations of myeloma patients compared to zoledronic acid [12,13]. However, side effects such as osteonecrosis of the jaw enhanced by both bisphosphonates and denosumab [12,14], as well as the need for new agents able to promote osteoblast-induced bone formation in MM, necessitate the proper understanding of the molecular background of MMBD and the detection of new molecular targets with possible therapeutic value [15,16].

MicroRNAs (miRNAs) constitute a class of small non-coding RNAs, bearing regulatory potency. They are small single-stranded molecules, about 21 nucleotides long that are transcribed as large primary RNA transcripts and are afterward processed through a two-step procedure by the RNases DROSHA and DICER. miRNAs regulate translation through RNA interference. A mature miRNA targets specific mRNAs through binding to regions of homology found most frequently in the 3′ untranslated region (UTR) of the target mRNA, but also the 5′ UTR and the coding region. Following binding in the 3′ UTR, translation is negatively regulated through inhibition of translation of the target mRNA or its degradation. However, enhancing the translation has been also reported upon binding to the 5′ UTR [17]. A single miRNA displays multiple mRNA targets [18]. miRNA expression is deregulated in a variety of cancers [19], and hematological malignancies [20], including multiple myeloma [21]. Their role can be characterized as either oncogenic or oncosuppressive. In human malignancies, deregulation of miRNA expression can be a result of several mechanisms including gene amplification or deletion, impaired biogenesis, or epigenetic changes. miRNAs in malignant cells have been witnessed to target pathways related to proliferation and apoptosis and also promote metastasis and induce angiogenesis, providing them with a survival benefit [22]. Moreover, miRNAs seem to be implicated in bone metabolism and bone disease by regulating the major pathways of the bone-remodeling process. Some of them have been witnessed to play even a dual role in bone disease, by affecting both osteoblast, and osteoclast function [23,24]. Based on such observations, scientific research focused on investigating miRNAs implicated in normal bone remodeling, assuming that their impaired expression may play a role in MMBD In this review, we provide concise information regarding the molecular pathways with a significant role in MMBD and the miRNAs implicated in their regulation. Moreover, we discuss their utility as molecular biomarkers and highlight the putative usage of miRNAs as novel molecular targets for targeted therapy in MMBD.

## 2. miRNAs Interacting with the Pro-Inflammatory Microenvironment of the MM Bone Marrow Niche Responsible for MMBD

Interactions between malignant MM cells and other components of the bone marrow microenvironment are crucial for MM development. An inflammatory immune-suppressive bone marrow microenvironment is responsible for disease progression, drug resistance, and bone disease [25,26]. Several cytokines seem to play an important role in MMBD. In MM bone marrow, marrow infiltrating lymphocytes possess a Th17 phenotype, which are CD4+ T cells that secrete IL-17. More specifically, in the MM microenvironment, IL6 and TGF-β produced by MM cells promote Th17 polarization, while IL-17 (also known as IL17A) produced by Th17 cells promotes tumor progression and MMBD [27]. Preclinical MM models demonstrated a significant role of IL-17 in osteoclast activation and lytic bone lesions formation. It is important to mention that when marrow infiltrating lymphocytes were shifted towards Th1 differentiation a significantly reduced formation of mature osteoclasts was observed [28]. Given those facts, an anti-IL-17A monoclonal antibody was used in MM mouse models and led to reduced growth and survival of MM cells and reduced bone destruction [29]. miR-21-5p promotes Th17 differentiation by inhibiting *PIAS3* [30], as well as by targeting and downregulating SMAD7 [31]; PIAS3 is responsible for inhibiting STAT3 signaling, thus its inhibition leads to sustained STAT3 signaling, which is critical for Th17 differentiation [30]. Th17 cells from MM patients express higher levels of miR-21-5p than those from normal controls. Moreover, miR-21-5p levels in Th17 cells from MM patients with active MMBD are higher than those in Th17 cells from MM patients without MMBD. Inhibition of miR-21-5p in naïve T-cells in vitro negatively regulated Th17 differentiation. In vivo, inhibition of miR-21-5p attenuated MM cell growth and osteoclast action. Integrated RNA sequencing and proteomics/phospho-proteomics analysis revealed that early inhibition of miR-21-5p in Th cells under Th17 polarizing conditions leads to a switch in Th1/2 like polarized cells, which are effector T cells mediating cell-mediated and humoral immune responses respectively [32].

MM cells secrete cytokines that promote bone resorption by osteoclasts [33]. CCL3, also known as MIP1A, is a chemokine secreted by MM cells [34]; high levels of MIP1A in MM cells and the serum of MM patients correlate with the severity of bone disease as well as with worse prognosis [35]. CCL3 promotes osteoclastogenesis by augmenting the effects of RANKL and IL6 in mature osteoclast formation, while it also inhibits bone formation by osteoblasts through downregulation of RUNX2, a transcription factor regulating the expression of genes with an important role in osteoblastogenesis [36], and osterix, a transcription factor highly abundant in osteoblasts and required for bone formation [37,38]. As proposed by a recent study, miR-29b-3p is downregulated in dendritic cells exposed to MM plasma cells, and this deregulation is linked to MM progression. CCL3 and its downstream targets were hindered upon miR-29b-3p overexpression, similarly to other molecules participating in pro-inflammatory pathways in tumor-associated dendritic cells, such as NF-κB and STAT3 [39]. NFKB1 promotes MM survival by upregulating antiapoptotic proteins such as BCL2 and MCL1 and simultaneously by downregulating pro-apoptotic proteins such as NOXA (also known as PMAIP1) and BBC3 (also known as Puma) [33]. Additionally, NF-κB signaling has been associated with increased bone resorption [40] and has been found to promote MM cell survival, by acting synergistically with STAT3. Moreover, miR-29b-3p enforced overexpression led to a decrease in IL-23 (also known as IL23A) levels and prohibited Th-17 polarization by dendritic cells in vitro and in vivo, leading to reduced cell growth and osteoclast-dependent bone destruction [39]. 

## 3. miRNAs as Signaling Pathway Regulators in MMBD

### 3.1. miRNAs Implicated in the Regulation of the RANK/RANKL Signaling Pathway

The RANK/RANKL pathway plays a major role in osteoclastogenesis and is also implicated in MMBD. RANK receptor (also known as TNFRSF11A) is a member of the TNF superfamily and is expressed on the surface of pre-osteoclasts. RANKL (also known as TNFSF11) is a cytokine expressed mainly in osteocytes, as well as in bone marrow stromal cells (BMSCs) and activated lymphocytes. Binding of RANKL to RANK leads to fusion of pre-osteoclasts and subsequently to the formation of mature osteoclasts, capable of initiating bone resorption. Osteoprotegerin (OPG; also known as TNFRSF11B) is also a member of the TNF superfamily that is secreted by osteoblasts and serves as a decoy receptor for RANKL. This means that OPG can bind RANKL and consequently inhibit RANKL binding to RANK. The balance between RANKL and OPG regulates osteoclast activity [41].

In MM the RANK/RANKL pathway is deregulated, leading to increased bone resorption [42]. In the MM bone marrow microenvironment, the balance between RANKL and OPG is disturbed; thus OPG is downregulated, while RANKL is overexpressed [43]. In brief, MM cells express transmembrane and soluble RANKL, thus promoting osteoclast differentiation and bone destruction [44]. Malignant plasma cells also promote RANKL production by osteoblasts and BMSCs by secreting PTHrP (also known as PTHLH), which acts in a paracrine way in the bone marrow niche. Additionally, ΜΜ cells secrete DKK1, an inhibitor of the WNT signaling pathway, leading to a decrease in OPG expression, as OPG regulation is mediated through the WNT pathway [45,46]. In addition to that, ΜΜ cells reduce OPG levels by producing SDC1, a transmembrane proteoglycan that binds to OPG and leads to its degradation by MM cells [47]. 

**Figure 2:**
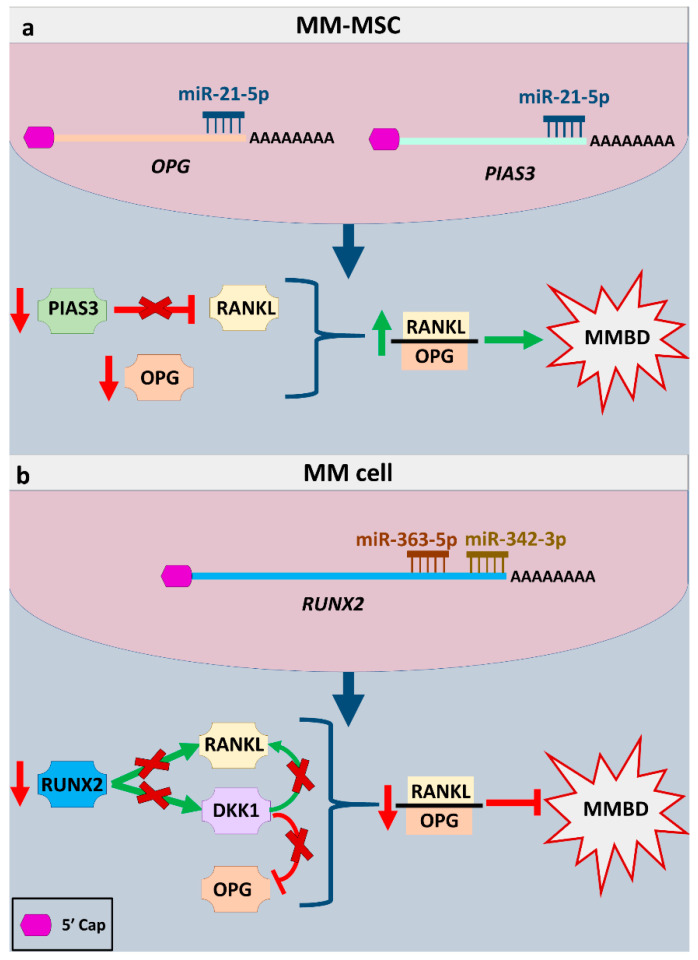
Two typical examples of miRNAs implicated in MMBD through the regulation of specific pathways. (**a**) miRNA action leads to an increase of the RANKL/OPG ratio and promotes MMBD through inhibiting *OPG* and *PIAS3*, a RANKL inhibitor; (**b**) miRNA action leads to the decrease of this ratio through inhibiting *RUNX2*, and its downstream targets, RANKL and DDK1, which is an OPG inhibitor. Abbreviation: MM-MSC, multiple myeloma mesenchymal stem cell.

This imbalance in RANKL/OPG ratio is also a result of miR-21-5p function; miR-21-5p, whose oncogenic role has been variously described, was found to be upregulated in mesenchymal stem cells from MM patients (MM-MSCs) compared to normal mesenchymal stem cells (MSCs). Experimental data supported that miR-21-5p is implicated in MMBD, and its inhibition restores the RANKL/OPG balance in MM-MSCs, resulting in increased OPG secretion and reduced bone resorption by mature osteoclasts. This is explained by the fact that *OPG* is a direct target of miR-21-5p [30]. Moreover, miR-21-5p inhibits *PIAS3*, which negatively regulates RANKL [48], leading to further RANKL upregulation and subsequently imbalance in RANKL/OPG ratio [30]. This information is graphically illustrated in Figure 2a.

Another miRNA affecting the RANK receptor pathway is miR-29b-3p. Normally, miR-29b-3p is downregulated during osteoclast differentiation, similarly to MM-associated dendritic cells [39]. Its overexpression during osteoclastogenesis led to reduced osteoclast-dependent bone destruction, due to reduced RANK expression on osteoclast surface and subsequent limited osteoclast response to RANKL. Regarding the mechanistic action of miR-29b-3p, it exerts its function by directly targeting the transcription factor *FOS* and *MMP2* metalloproteinase. MMP2 metalloproteinase is overexpressed in the bone marrow of MM patients and is associated with MM progression [49]. FOS represents a vital transcription factor in osteoclastic differentiation and forms a regulatory positive feedback loop with RANK and NFKB1 [50]. Moreover, FOS induces the expression of major transcription factors for osteoclasts that regulate the expression of osteoclast key genes, such as CTSK, MMP9, and ACP5 (also known as TRAcP) [51]. These data explain the lower intracellular levels of all these vital enzymes for osteoclast function. Intriguingly, miR-29b-3p overexpressing osteoclasts retained their impaired activity, even when co-cultured with MM cells, despite the strong pro-osteoclastic stimuli provided by them, indicating the power of its effect. These data, along with the analytically described function of miR-29b-3p in dendritic cells [39], enhance the hypothesis that miR-29b-3p has a strong therapeutic potency.

### 3.2. miRNAs Implicated in the Regulation of the WNT Signaling Pathway

WΝΤ signaling pathway is implicated in a variety of malignancies, as well as in the bone formation and bone disease. WΝΤ signaling pathway is stimulated by binding of a WΝΤ canonical ligand to a receptor complex constituted by a Frizzled receptor and a WNT co-receptor lipoprotein (LRP5 or LRP6). In the inactivated state, cytoplasmic β-catenin (also known as CTNNB1) is bound to a “destruction complex” constituted by APC, AXIN2 (also known as CSNK1A1), and GSK3Β serine-threonine kinase, that leads to phosphorylation of β-catenin and as result ubiquitination and proteasomal degradation of the molecule. When a canonical WNT ligand binds to the receptor complex, AXIN2 interacts with DVL and is translocated to LRP5/6 at the membrane, where a complex is formed. As a result, phosphorylation of β-catenin by GSK3B is blocked. Cytoplasmic β-catenin levels increase and the molecule is translocated to the nucleus, where it displaces TLE1 (also known as Groucho) and interacts with TCF and LEF transcription factors, thus increasing transcription of their target genes and promoting bone formation. Regarding the non-canonical WNT signaling, it is β-catenin independent [52,53]. In bone metabolism, WNT signaling promotes differentiation of MSCs to osteoblasts and supports the survival of osteoblasts [54,55]. 

The WNT signaling pathway is implicated in MMBD. MM cells and osteocytes express molecules that act as WNT inhibitors, thus leading to decreased bone formation and increased bone resorption. These molecules are sclerostin (also known as SOST), DKK1, and secreted proteins SFRP2 and FRZB [56]. DKK1, a key molecule in MMBD, binds to the co-receptor LRP5/6 in conjunction with the transmembrane proteins KREMEN1 and KREMEN2 leading to internalization of LRPs and inactivation of WNT signaling [57]. Additionally, DKK1 inhibits osteoblastogenesis by inhibiting autocrine WNT signaling, which is necessary for differentiation of the osteoblast lineage, and as a result, undifferentiated MSCs produce IL6, which in turn promotes the proliferation of MM cells that secrete more DKK1 [58]. Moreover, DKK1 indirectly promotes osteoclastogenesis by increasing the RANKL/OPG ratio, since RANKL and OPG production in osteoblasts is regulated by the WNT signaling pathway [46], and so does sclerostin [59]. Sclerostin is produced by osteocytes and is a cysteine-knot containing protein that binds to LRP5/6 and inhibits WNT signaling activation in osteoblast lineage cells. Sclerostin promotes apoptosis of mature osteoblasts by activating the caspase pathway and inhibits BMP-mediated mineralization in osteoblasts by preventing BMPs, which are TGF-β superfamily ligands that participate in bone formation, from binding to their receptors [60,61] The role of BMP-mediated signaling will be analytically discussed in the respective section. MM cells secrete sclerostin and elevated circulating levels of this molecule correlate with adverse prognosis of MM patients [62,63].

A miRNA implicated in the regulation of the canonical WNT pathway is miR-203a-3p. miR-203a-3p is implicated in the regulation of osteogenesis possibly through inhibition of the WNT3A/β-catenin pathway. In MM-MSCs its expression levels were downregulated compared to normal MSCs. Upon osteogenic induction, miR-203a-3p was downregulated in normal MSCs, while no change was observed in MM-MSCs. Inhibition of miR-203a-3p led to increased expression levels of WNT3A, β-catenin, and GSK3B, genes implicated in the WNT/β-catenin pathway and as a result to increased osteoblast differentiation. This means that miR-203a-3p inhibition may induce osteogenesis by activating the canonical WNT signaling pathway [64]. 

Regarding the non-canonical WNT signaling pathway, most studies focus on the aforementioned RUNX2 [36]. MM cells inhibit osteoblast differentiation by blocking RUNX2 activity in MM-MSCs, although CYR61 upregulates RUNX2 in MM models, promoting osteogenic differentiation [65]. In vivo, RUNX2 expression levels were lower in the bone marrow biopsies from MM patients with bone disease compared to those without bone disease [66]. However, high levels of RUNX2 expressed by MM cells were correlated with advanced disease characteristics and poor prognosis [67]. This comes in line with the fact that RUNX2 facilitated both osteoblastogenesis and osteoclastogenetic signals in mesenchymal progenitor cells [68].

Gowda et al. showed that miR-342-3p and miR-363-5p regulate MM progression and also have a role in MMBD by targeting individually or synergistically and regulating *RUNX2* expression. Those two molecules were downregulated in MM plasma cells compared to plasma cells from healthy donors, while RUNX2 levels were upregulated. miR-342-3p and miR-363-5p upregulation in MM cells resulted in lower RUNX2 expression and subsequent lower expression of RUNX2 target genes, such as RANKL and DKK1, which promote MM proliferation and MMBD, as shown in Figure 2b. Moreover, RUNX2 downstream AKT/β-catenin/Survivin (also known as BIRC5) signaling pathway was suppressed, leading to deregulation of bone formation-related TCF and LEF transcription factors and consequently tumor suppression [52,69]. MM cells overexpressing miR-342-3p and miR-363-5p injected in mice resulted in decreased MM cell growth and led to a decreased number of osteoclasts and increased number of osteoblasts, as well as increased antitumor immunity in an in vivo model [69], enhancing the hypothesis that they are able to suppress MMBD.

### 3.3. miRNAs Implicated in the Regulation of the TGF-β/SMAD and BMP/SMAD Signaling Pathways

SMAD family proteins are intracellular signal transducers of TGF-β superfamily receptors. TGF-β family receptors consist of a complex of two type I and two type II receptors with serine/threonine kinase activity. Following ligand activation, type I receptors activate SMADS by phosphorylating their two C-terminal serine residues. In mammals, 8 types of SMAD proteins are detected. These are the receptor-activated SMADS (R-SMADS; SMAD1,2,3,5,8), the common-mediator SMAD (SMAD4), and the inhibitory SMADS (I-SMADS; SMAD 6,7). After activation, R-SMADS form complexes with SMAD4 and insert the nucleus, where they interact with transcription factors and regulate target genes. I-SMADS act by blocking R-SMADS activation or formation of the I-SMAD/SMAD4 complex [70]. 

The role of TGF-β signaling in osteogenesis is rather controversial, as it has been shown to both facilitate and inhibit osteoclast differentiation [71]; Regarding osteoblasts, TGF-β-mediated signaling is vital for the differentiation of the osteoblast progenitors, but on the contrary, inhibits osteoblast maturation [72]. TGF-β has been reported to suppress osteoblast differentiation of MM-MSCs by inhibiting BMP2 signaling and suppressing the canonical WNT pathway [73]. Specifically, TGF-β is produced by osteoblasts and osteocytes and is found in the bone matrix in an inactive form. Bone resorption by osteoclasts releases activated TGF-β. However, SMAD3, which is a downstream component of the TGF-β signaling [74], is required for osteoblast differentiation, as its deficiency leads to impaired bone formation [75]. Additionally, SMAD3-deficient mice displayed abnormal skeletal development [76]. miR-221-5p is a miRNA that contributes to the reduced osteogenic capacity of MM-MSCs by regulating osteogenesis through interacting with *SMAD3*. Following osteoblast induction, miR-221-5p levels are lower in normal donor MSCs, compared to MM-MSCs. Inhibition of miR-221-5p expression resulted in the promotion of osteoblast differentiation of MM-MSCs by upregulation of *SMAD3*, which is a direct target of miR-221-5p [77]. As SMAD3 possesses an important role in osteogenesis as already explained, its downregulation due to miR-221-5p action causes reduced bone formation and osteopenia by dysregulation of osteoblast differentiation [75]. Additionally, inhibition of miR-221-5p led to the activation of the PI3K/AKT/mTOR signaling pathway, which is implicated in the survival, proliferation, and migration, thus stimulating osteogenic differentiation of MM-MSCs [77].

BMPs, which are members of the TGF-β superfamily and have been shown to play an also controversial role in MMBD [70]. More specifically, transcriptomic profiling experiments showed that BMP-mediated signaling inhibition suppressed MMBD [78]. However, another study showed that BMP2 promotes osteoblastogenesis [79], while MM cells secrete hepatocyte growth factor, which in turn inhibits BMP-induced osteoblast differentiation [80]. Furthermore, the downstream mediators of BMP signaling, namely SMAD1, SMAD5, and SMAD8 (also known as SMAD9) are essential for bone formation, as their deficiency in mice leads to lethal chondrodysplasia [81]. miR-135b-5p was found to participate in this network, as it directly targets and downregulates SMAD5, leading to impaired osteogenic potential, by causing reduced ALPL activity and decreased expression of the osteogenic markers BSP, COLA1, and OPN. MM-MSCs show higher miR-135b-5p levels in comparison with MSCs from normal donors. Co-culture of normal donor MSCs with MM cells resulted in overexpression of miR-135b-5p in normal donor cells, indicating that MM cells secrete soluble factors capable to upregulate miR-135b-5p expression in normal MSCs. This interaction could be mediated by extracellular vehicles; this aspect will be analytically described in the respective section. Treatment of MM-MSCs with a miR-135b-5p inhibitor improved their osteogenic potential indicating a possible new molecular therapeutic approach for MM-related bone disease [82]. Another miRNA that directly targets a SMAD protein is the aforementioned miR-203a-3p, which regulates osteogenic differentiation of MM-MSCs not only via regulation of the canonical WNT pathway but also by targeting *SMAD9*. Although miR-203a-3p levels are lower in MM-MSCs compared to normal donor MSCs, a decrease in the expression levels of miR-203a-3p was observed upon osteogenic induction in normal MSCs, while no significant change was observed in MM-MSCs. Downregulation of miR-203a-3p in MM-MSCs led to the promotion of osteogenesis by upregulation of *SMAD8*, followed by an increase in the mRNA expression levels of osteoblastic differentiation markers such as ALPL, OC, OPN [64]. Interestingly, crosstalk between TGF-β/SMAD and WNT pathways has been reported [83]; a study suggested that these pathways cooperatively regulate TCF and LEF transcription factors [84], both implicated in bone formation [52]. Therefore, it is possible that miR-203a-3p exerts its function in MMBD by affecting both pathways.

Last, miR-34a-5p, a miRNA with an established oncosuppressive role, is also implicated in MMBD through the regulation of SMAD proteins. Its upregulation in MM stem cells leads to decreased cell proliferation in vitro, as well as reduced tumor growth and fewer osteolytic lesions in vivo. miR-34a-5p exerts its tumor-suppressive action by targeting and downregulating TGIF2 [85]; TG1F2 constitutes a transcriptional regulator that either binds directly to *SMAD* genes, recruiting histone deacetylases, or interacts with TGF-β-activated SMADs, repressing SMAD-mediated signaling [86]. In this way, miR-34a-5p promotes the functional SMAD formation and advocates their efficient function, consequently suppressing the formation of osteolytic lesions [85]. Further investigation regarding the SMAD-mediated signaling is required, in order to uncover its role in the development of MMBD. The elucidation of additional miRNAs implicated in this pathway could facilitate the deciphering of the interactions within this pathway and shed light on its implication in osteoblastogenesis and osteoclastogenesis.

### 3.4. miRNAs Implicated in the NOTCH Signaling Pathway

The NOTCH pathway consists of 4 transmembrane receptors (NOTCH1-4) and 2 types of ligands, the jagged ligands (JAG1, JAG2) and the delta-like ligands (DLL1, DLL3, DLL4). NOTCH receptors from the receiving cells bind to their ligands expressed on the membrane of the adjacent sending cells. Upon binding of a ligand to a NOTCH receptor, protease ADAM cleaves the extracellular domain of the NOTCH receptor, while the γ-secretase complex cleaves and releases in the cytoplasm the intracellular domain of NOTCH (ICN). ICN is translocated to the nucleus and promotes transcription of the target genes HES and HEY. NOTCH signaling is implicated in osteoclastogenesis induced by MM cells. MM cells express NOTCH1,2,3 receptors that bind to their ligands on the same cell, forming a homotypical interaction, or on adjacent cells in the bone marrow microenvironment, forming a heterotypical interaction. NOTCH activation leads to increased expression of RANKL by MM cells. Moreover, MSCs also express NOTCH receptors and can interact with MM cells resulting in increased expression of RANKL [87].

miR-223-3p has been shown to exert several regulatory roles in bone metabolism, mainly by regulating molecules with a critical role in the differentiation of both osteoclasts and osteoblasts [88], and has been associated with NOTCH pathway. miR-223-3p expression in MM-MSCs co-cultured with MM cells was reduced in a cell-adhesion-dependent way. Additionally, miR-223-3p expression levels were decreased upon JAG2 upregulation. miR-223-3p repression led to increased expression of tumor supportive cytokines VEGF and IL6 and inhibition of the osteogenic potential of MM-MSCs, as indicated by reduced expression of RUNX2 and OPN, as well as reduced calcification and ALPL activity [89]. Notch signaling inhibition in MM-MSCs led to an increase in miR-223-3p levels and a decrease in VEGF and IL6. These data imply a Notch-dependent expression and function of miR-223-3p in MM-MSCs; however, the exact mechanism has not been deciphered yet.

The main miRNAs implicated in pathways related to MMBD are summarized in Table 1.

### 3.5. miRNAs Regulating the Expression of Other Genes Involved in Osteochondrogenesis

miR-138-5p is a molecule, whose role in osteogenic differentiation and MMBD has been extensively studied. miR-138-5p is upregulated in MM-MSCs and in MM cells compared to MSCs from normal donors and normal plasma cells respectively. There is a possible interplay between MM cells and MSCs at the bone marrow microenvironment since the co-culture of MSCs from normal donors with MM cells led to overexpression of miR-138-5p in MSCs. Inhibition of miR-138-5p promotes osteogenic differentiation of MM-MSCs in vitro. Moreover, the total number of endosteal osteoblastic lineage cells and bone formation rate were increased in in vivo models upon miR-138-5p inhibition. miR-138-5p exerts its role in osteogenic differentiation of MM-MSCs through targeting and downregulating three target genes: ROCK2, TRPS1, and SULF2 [90]. All those three genes are involved in osteochondrogenesis. ROCK kinases are kinases activated by the Rho family of small GTPases. Activation of the RhoA-ROCK pathway in MSCs promotes osteoblast differentiation [91,92]. TRPS1 gene encodes a GATA-transcription factor that regulates transcription of genes involved in proliferation, differentiation, and apoptosis of chondrocytes. TRPS1 has been also identified as a target of a series of miRNAs that regulate osteogenesis [93,94]. Extracellular sulfatase SULF2 is an enzyme implicated in the differentiation of many tissues. SULF2 is overexpressed in bone-forming osteoblasts, as well as in hypertrophic chondrocytes during development and bone fracture healing [95]. It is obvious that miR-138-5p upregulation in MM-MSCs leads to impaired osteogenesis as a result of the downregulation of key genes implicated in that process.

## 4. Extracellular Vesicle-Associated miRNAs Implicated in MMBD

Extracellular vesicles (EVs) are a heterogeneous group of membranous vesicles produced by various cell types. They are divided into two categories depending on size and origin: exosomes (50–150 nm), which derive from the endosomal system, and microvesicles (50 nm–1 μm), which arise from the budding of the plasma membrane. EVs contain lipids, proteins, DNA, coding, and non-coding RNA and they represent a mechanism of intercellular communication [96]. EVs play an important role in the bone marrow microenvironment in MM, supporting communication between plasma cells and other cell types such as stromal, endothelial, immune cells, and MSCs, thus promoting MM progression [97,98,99,100]. Many studies show that MM-derived EVs play an important role in MMBD causing an imbalance between osteoclast-induced bone resorption and osteoblast mediated bone formation. MM cells secrete EVs that are internalized by primary osteoclasts leading to expression of osteoclast markers. Treatment of pre-osteoclasts with MM-derived EVs, led to their differentiation in mature multinuclear osteoclasts with strong resorptive ability [101]. Further studies revealed that the EGFR ligand AREG, which was highly expressed in MM EVs, led to the activation of the EGFR pathway, a key pathway implicated in growth, survival, and proliferation, in pre-osteoclasts, thus inducing osteoclast differentiation [102]. Apart from their effect on osteoclast differentiation, MM EVs also exert an inhibitory role in osteoblast formation. One of the possible mechanisms is by reducing the expression of RUNX2, osterix, and OCN and increasing APE1 and NFKB1. Another study proposed that MM-EVs transfer DKK1 to osteoblasts leading to reduced RUNX2 and osterix [102,103,104,105]. 

**Figure 3:**
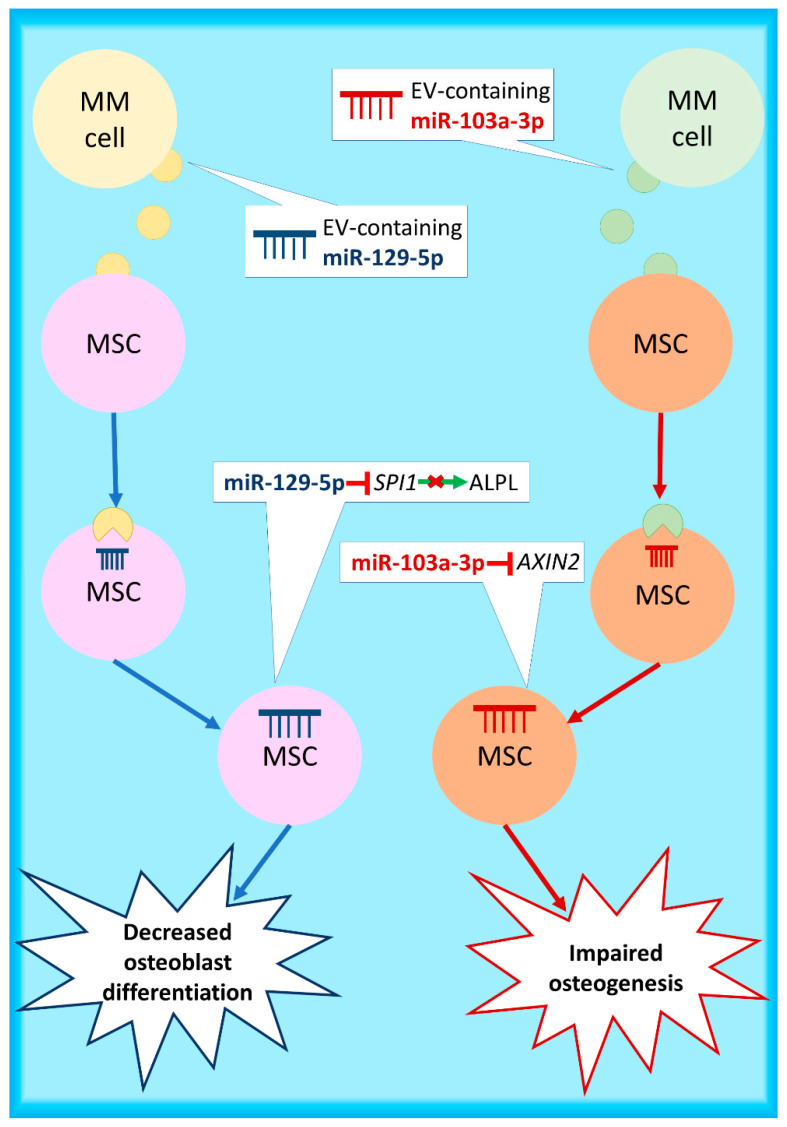
MM-derived extracellular vesicles (EVs) containing miRNAs can be internalized from MSCs, leading to diminished osteogenic potential.

Increasing evidence indicates miRNAs as the cargo of EVs responsible for their impact on target cells. miRNAs contained in EVs seem to regulate gene expression on the recipient cells [106,107]. miRNAs most frequently incorporated in EVs share an extra-seed sequence, the hEXO motif. The RNA binding protein SYNCRIP binds to the hEXO motif sorting those miRNAs into EVs [108]. Several studies have demonstrated that miRNAs are contained in MM-derived EVs [97,109,110].This mechanism could also explain the overexpression of miR-138-5p and miR-135b-5p in normal donor MSCs upon their co-culture with MM cells [82,90]. In this context, studies revealed that miRNAs implicated in MMBD are found in MM-EVs. miR-129-5p abundance is significantly higher in EVs from MM patients compared to those deriving from smoldering MM (sMM) patients implying its putative role as a miRNA responsible for EV-mediated MMBD. MM-EV-derived miR-129-5p targets and downregulates *SPI1*, a transcriptional factor that promotes osteoblast differentiation by enhancing transcription of ALPL, a marker of osteogenic differentiation. MSCs treated with MM-EVs internalized them, and as a result, miR-129-5p levels in MSCs were upregulated, and consequently osteogenic differentiation was blocked [111]. Another miRNA showing a such function is miR-103a-3p; this miRNA has been shown to be significantly upregulated in serum exosomes of MM patients compared to exosomes of sMM patients and healthy individuals [112]. Within this framework, another study showed that miR-103a-3p was upregulated in MSCs transfected with MM-EVs and resulted in impaired osteogenesis in vitro, as well as severe MMBD in vivo. Interestingly, the same study revealed that the levels of CD138+ EVs in the peripheral blood of newly-diagnosed MM patients correlated positively with the number of bone lesions [105]. The expression of miR-103a-3p has been reported to be regulated by the receptor of PTHrP, namely PTHR1; in osteosarcoma, miR-103a-3p inhibits *AXIN2* [113], a key component of the WNT signaling pathway, as analytically explained in the respective section. It is possible that EV-derived miR-103a-3p exerts this function in MM-MSCs, leading to severe MMBD. These data are graphically described in Figure 3. 

## 5. miRNAs as Biomarkers in MMBD

The availability in patients’ body fluids, the small size, and the ability of easy detection have advocated the consolidation of small non-coding RNAs as putative molecular biomarkers for various diseases, including hematological malignancies [20,114,115,116,117]. Due to their implication in the molecular background of MMBD, some studies have attempted to propose specific miRNAs as candidate biomarkers in MMBD.

In a large cohort of MM patients, serum miR-214-3p levels were higher in patients with bone disease compared to those without MMBD. Furthermore, circulating miR-214-3p levels in the serum of patients correlated with the extent of bone disease. miR-214-3p levels have also been correlated with adverse prognosis in the same study. When these patients were treated with bisphosphonates, a significant increase in progression-free survival and OS was observed [118]. The severe bone disease observed upon miR-214-3p overexpression can be explained by the fact that miR-214-3p derived by MM exosomes has been shown to target *PTEN* [109]. PTEN phosphatase is a key component of the PI3K/AKT signaling pathway, which stimulates osteogenic differentiation, as already explained [77]. The aforementioned results indicate that miR-214-3p can serve as a biomarker able to detect MM bone disease, as well as a prognostic marker for MM patients, able to guide therapeutic approaches.

As miR-135b-5p played a significant role in MMBD by being implicated in the BMP/SMAD pathway [82], the same study attempted to propose miR-135b-5p as a putative biomarker able to distinguish MM patients with bone disease from those without bone disease. miR-135b-5p expression levels were higher in patients with MMBD compared to those without. Furthermore, miR-135b-5p levels also correlated with the severity of MMBD [118].

Last, a recent study investigated the expression of miR-181a-5p in MM patients and its correlation with clinical characteristics of the disease. miR-181a-5p levels were evaluated in peripheral blood (serum) and bone marrow mononuclear cells from MM patients. A positive correlation was observed between miR-181a-5p expression and bone injury in MM patients [119]. This correlation can be explained by the fact that miR-181a-5p has been shown to promote MM progression, by contributing to the downregulation of cyclin D1 (*CCND1*) [120], which is also negatively regulated in MMBD due to the deregulation of the WNT signaling pathway [121].

## 6. Conclusions and Future Perspectives

MMBD is a result of deregulation in multiple signaling pathways. Preclinical studies indicate that miRNA regulation is implicated in the majority of them. These data present a novel perspective regarding targeted treatment in MMBD. Although well-established anti-myeloma regimens may improve bone metabolism [122,123,124,125], bone-targeted therapies are essential components of the therapeutic strategy [11]. Denosumab, a fully human IgG2 monoclonal antibody that targets RANKL is a typical paradigm of targeted treatment in MMBD [126]. Following denosumab, the scientific community turned towards targeting key molecules implicated in bone disease. This tendency is forced by the unmet need to find therapeutic targets able to promote osteoblast-induced bone formation in MM patients. It is true, that although many effective therapeutic options able to reduce osteoclast activity are available for the management of MMBD, molecules able to induce osteoblast activity in MM are still not available in clinical practice. One such example is anti-sclerostin antibodies. Sclerostin is a regulator of osteoblastic activity that inhibits the WNT/β-catenin pathway. Anti-sclerostin antibodies tested in xenograft MM models exhibited promising results [127,128]. Another example of targeted treatment with a positive effect on osteoblast function is DKK1 antagonists. As already mentioned above, DKK1 is an important molecule causing dysfunction of osteoblasts in MMBD. Based on data coming from preclinical studies researchers evaluated a monoclonal anti-DKK1 antibody, BHQ880 in combination with zoledronic acid in the relapsed and refractory setting in a phase I/II clinical trial. The combination exhibited increased bone strength at the hip and spine [129]. Recently, the scientific community shows increasing interest in miRNAs as therapeutic targets. Regarding malignancy, miRNA-targeted therapy consists of inhibition of oncomiRs and upregulation of tumor-suppressor miRNAs. Inhibition of oncogenic miRNAs can be achieved by antagomiRs, which are antisense miRNA molecules, or by miRNA-sponges (e.g., circular RNAs [130]) that are molecules that act as competing endogenous RNAs and thus are able to bind the miRNA and prevent it from binding to the target mRNA. Tumor-suppressor miRNAs can be therapeutically replaced by using viral and non-viral vectors and miRNA mimics [21]. Regarding MMBD there are many promising molecules that can serve as targets for miRNA targeted therapy. For example, MM-MSCs treated with a miR-135b-5p inhibitor in vitro regained their osteogenic potential, proposing miR-135b-5p as a possible new targeted therapy [82]. Moreover, scientific data support that miR-29b-3p regulates both the RANKL pathway and the pro-inflammatory MM bone marrow milieu, thus constituting an attractive candidate for targeted therapy for MM and the associated bone disease [39,51]. Unfortunately, there are still some limitations that make miRNA-targeted treatment challenging. Some of them are impaired stability of those molecules in the human body, poor tissue penetration, off-target effects, and severe immune-related responses [131]. miR-34a-5p is an established tumor suppressor microRNA. The first clinical trial using a miRNA-based treatment for malignancy used liposomes containing miR-34a-5p as a therapeutic molecule for patients with advanced solid cancers. Although the trial was primarily terminated due to serious immunologic reactions, a dose-dependent regulation of miR-34a-5p target genes was observed, indicating that miRNA based target therapy is possibly an effective treatment option, given that the limitations mentioned above will be overcome [132]. It is obvious that miRNA-based treatment for malignancy is the future of targeted therapies. As extensively described above, a great number of miRNAs are implicated in MMBD and can serve as target molecules for new treatments that could be able not only to prevent osteoclast-induced bone destruction but also to enhance bone formation and give MM patients quality of life benefit.

## Figures and Tables

**Table 1:** MicroRNAs (miRNAs) implicated in pathways affecting multiple myeloma bone disease (MMBD).

miRNA	Pathway	Target	Effect on Bone Metabolism	Reference
miR-21-5p	RANK/RANKL	*OPG; PIAS3*	Regulation of the RANKL/OPG balance	[30]
miR-29b-3p		*FOS; MMP2*	Reduction of osteoclast-dependent bone destruction	[51]
miR-221-5p	TGF-β/SMAD	*SMAD3*	Dysregulation of osteoblast differentiation	[77]
miR-34a-5p	BMP/SMAD	*TGIF2*	Reduction of tumorigenesis and lytic bone lesions	[85]
miR-135b-5p		*SMAD5*	Impairment of the osteogenic potential of MSCs	[82]
miR-203a-3p	BMP/SMAD	*SMAD* *8*		[64]
	WNT/β-catenin	–		
miR-342-3p; miR-363-5p	Non-canonical WΝΤ	*RUNX2*	Increase of osteoclast and reduction of osteoblast activity	[69]
miR-223-3p	NOTCH	–	Impairment of the osteogenic potential of MSCs	[89]

Abbreviation: MSC, mesenchymal stem cell.

## Data Availability

Not applicable.

## References

[1] Kumar S.K., Rajkumar V., Kyle R.A., Van Duin M., Sonneveld P., Mateos M.-V., Gay F., Anderson K.C. (2017). Multiple myeloma. Nat. Rev. Dis. Prim..

[2] Rajkumar S.V., Dimopoulos M.A., Palumbo A., Blade J., Merlini G., Mateos M.-V., Kumar S., Hillengass J., Kastritis E., Richardson P. (2014). International Myeloma Working Group updated criteria for the diagnosis of multiple myeloma. Lancet Oncol..

[3] Hillengass J., Usmani S., Rajkumar S.V., Durie B.G.M., Mateos M.-V., Lonial S., Joao C., Anderson K.C., García-Sanz R., Riva E. (2019). International myeloma working group consensus recommendations on imaging in monoclonal plasma cell disorders. Lancet Oncol..

[4] Gavriatopoulou M., Βoultadaki A., Koutoulidis V., Ntanasis-Stathopoulos I., Bourgioti C., Malandrakis P., Fotiou D., Migkou M., Kanellias N., Eleutherakis-Papaiakovou E. (2020). The role of low dose whole body ct in the detection of progression of patients with smoldering multiple myeloma. Blood Cancer J..

[5] Terpos E., Ntanasis-Stathopoulos I., Gavriatopoulou M., Dimopoulos M.A. (2018). Pathogenesis of bone disease in multiple myeloma: From bench to bedside. Blood Cancer J..

[6] Terpos E., Ntanasis-Stathopoulos I., Dimopoulos M.A. (2019). Myeloma bone disease: From biology findings to treatment approaches. Blood.

[7] Kyriazoglou A., Ntanasis-Stathopoulos I., Terpos E., Fotiou D., Kastritis E., Dimopoulos M.A., Gavriatopoulou M. (2020). Emerging insights into the role of the hippo pathway in multiple myeloma and associated bone disease. Clin. Lymphoma Myeloma Leuk..

[8] Terpos E., Ntanasis-Stathopoulos I., Christoulas D., Bagratuni T., Bakogeorgos M., Gavriatopoulou M., Eleutherakis-Papaiakovou E., Kanellias N., Kastritis E., Dimopoulos M.A. (2018). Semaphorin 4D correlates with increased bone resorption, hypercalcemia, and disease stage in newly diagnosed patients with multiple myeloma. Blood Cancer J..

[9] Terpos E., Morgan G., Dimopoulos M.A., Drake M.T., Lentzsch S., Raje N., Sezer O., García-Sanz R., Shimizu K., Turesson I. (2013). International Myeloma Working Group recommendations for the treatment of multiple myeloma–related bone disease. J. Clin. Oncol..

[10] Terpos E., Christoulas D., Gavriatopoulou M., Dimopoulos M. (2017). Mechanisms of bone destruction in multiple myeloma. Eur. J. Cancer Care.

[11] Terpos E., Zamagni E., Lentzsch S., Drake M.T., García-Sanz R., Abildgaard N., Ntanasis-Stathopoulos I., Schjesvold F., de la Rubia J., Kyriakou C. (2021). Treatment of multiple myeloma-related bone disease: Recommendations from the Bone Working Group of the International Myeloma Working Group. Lancet Oncol..

[12] Raje N., Terpos E., Willenbacher W., Shimizu K., García-Sanz R., Durie B., Legieć W., Krejčí M., Laribi K., Zhu L. (2018). Denosumab versus zoledronic acid in bone disease treatment of newly diagnosed multiple myeloma: An international, double-blind, double-dummy, randomised, controlled, phase 3 study. Lancet Oncol..

[13] Terpos E., Raje N., Croucher P., Garcia-Sanz R., Leleu X., Pasteiner W., Wang Y., Glennane A., Canon J., Pawlyn C. (2021). Denosumab compared with zoledronic acid on PFS in multiple myeloma: Exploratory results of an international phase 3 study. Blood Adv..

[14] Terpos E., Roodman G.D., Dimopoulos M.A. (2013). Optimal use of bisphosphonates in patients with multiple myeloma. Blood.

[15] Terpos E., Berenson J., Raje N., Roodman G.D. (2014). Management of bone disease in multiple myeloma. Expert Rev. Hematol..

[16] Terpos E., Ntanasis-Stathopoulos I. (2020). Controversies in the use of new bone-modifying therapies in multiple myeloma. Br. J. Haematol..

[17] Gu W., Xu Y., Xie X., Wang T., Ko J.-H., Zhou T. (2014). The role of RNA structure at 5′ untranslated region in microRNA-mediated gene regulation. Rna.

[18] Gebert L.F.R., Macrae I.J. (2019). Regulation of microRNA function in animals. Nat. Rev. Mol. Cell Biol..

[19] Acunzo M., Romano G., Wernicke D., Croce C.M. (2015). MicroRNA and cancer—A brief overview. Adv. Biol. Regul..

[20] Katsaraki K., Karousi P., Artemaki P., Scorilas A., Pappa V., Kontos C., Papageorgiou S. (2021). MicroRNAs: Tiny regulators of gene expression with pivotal roles in normal B-Cell development and B-Cell chronic lymphocytic leukemia. Cancers.

[21] Soliman A.M., Lin T.S., Mahakkanukrauh P., Das S. (2020). Role of microRNAs in diagnosis, prognosis and management of multiple myeloma. Int. J. Mol. Sci..

[22] Peng Y., Croce C.M. (2016). The role of MicroRNAs in human cancer. Signal. Transduct. Target..

[23] Shi K., Lu J., Zhao Y., Wang L., Li J., Qi B., Li H., Ma C. (2013). MicroRNA-214 suppresses osteogenic differentiation of C2C12 myoblast cells by targeting Osterix. Bone.

[24] Zhao C., Sun W., Zhang P., Ling S., Li Y., Zhao D., Pengfei Z., Wang A., Li Q., Song J. (2015). miR-214 promotes osteoclastogenesis by targeting Pten/PI3k/Akt pathway. Rna Biol..

[25] Rossi M., Botta C., Correale P., Tassone P., Tagliaferri P. (2013). Immunologic microenvironment and personalized treatment in multiple myeloma. Expert Opin. Biol..

[26] Görgün G.T., Whitehill G., Anderson J.L., Hideshima T., Maguire C., Laubach J., Raje N., Munshi N.C., Richardson P.G., Anderson K.C. (2013). Tumor-promoting immune-suppressive myeloid-derived suppressor cells in the multiple myeloma microenvironment in humans. Blood.

[27] Prabhala R.H., Pelluru D., Fulciniti M., Prabhala H.K., Nanjappa P., Song W., Pai C., Amin S., Tai Y.-T., Richardson P.G. (2010). Elevated IL-17 produced by Th17 cells promotes myeloma cell growth and inhibits immune function in multiple myeloma. Blood.

[28] Noonan K., Marchionni L., Anderson J., Pardoll D., Roodman G.D., Borrello I. (2010). A novel role of IL-17–producing lymphocytes in mediating lytic bone disease in multiple myeloma. Blood.

[29] Prabhala R.H., Fulciniti M., Pelluru D., Rashid N.U., Nigroiu A., Nanjappa P., Pai C., Lee S., Prabhala N.S., Bandi R.L. (2016). Targeting IL-17A in multiple myeloma: A potential novel therapeutic approach in myeloma. Leukemia.

[30] Pitari M.R., Rossi M., Amodio N., Botta C., Morelli E., Federico C., Gullà A., Caracciolo D., Di Martino M.T., Arbitrio M. (2015). Inhibition of miR-21 restores RANKL/OPG ratio in multiple myeloma-derived bone marrow stromal cells and impairs the resorbing activity of mature osteoclasts. Oncotarget.

[31] Murugaiyan G., Da Cunha A.P., Ajay A.K., Joller N., Garo L.P., Kumaradevan S., Yosef N., Vaidya V.S., Weiner H.L. (2015). MicroRNA-21 promotes Th17 differentiation and mediates experimental autoimmune encephalomyelitis. J. Clin. Investig..

[32] Rossi M., Altomare E., Botta C., Cantafio M.E.G., Sarvide S., Caracciolo D., Riillo C., Gaspari M., Taverna D., Conforti F. (2020). miR-21 antagonism abrogates Th17 tumor promoting functions in multiple myeloma. Leukemia.

[33] Hengeveld P.J., Kersten M.J. (2015). B-cell activating factor in the pathophysiology of multiple myeloma: A target for therapy?. Blood Cancer J..

[34] Terpos E., Politou M., Viniou N., Rahemtulla A. (2005). Significance of macrophage inflammatory protein-1 alpha (MIP-1α) in multiple myeloma. Leuk. Lymphoma.

[35] Terpos E., Politou M., Szydlo R., Goldman J.M., Apperley J.F., Rahemtulla A. (2003). Serum levels of macrophage inflammatory protein-1 alpha (MIP-1α) correlate with the extent of bone disease and survival in patients with multiple myeloma. Br. J. Haematol..

[36] Marie P.J. (2008). Transcription factors controlling osteoblastogenesis. Arch. Biochem. Biophys..

[37] Oyajobi B.O., Franchin G., Williams P.J., Pulkrabek D., Gupta A., Munoz S., Grubbs B., Zhao M., Chen D., Sherry B. (2003). Dual effects of macrophage inflammatory protein-1α on osteolysis and tumor burden in the murine 5TGM1 model of myeloma bone disease. Blood.

[38] Fu R., Liu H., Zhao S., Wang Y., Li L., Gao S., Ruan E., Wang G., Wang H., Song J. (2014). Osteoblast inhibition by chemokine cytokine ligand3 in myeloma-induced bone disease. Cancer Cell Int..

[39] Botta C., Cucè M., Pitari M.R., Caracciolo D., Gullà A., Morelli E., Riillo C., Biamonte L., Cantafio M.E.G., Prabhala R. (2018). MiR-29b antagonizes the pro-inflammatory tumor-promoting activity of multiple myeloma-educated dendritic cells. Leukemia.

[40] Abu-Amer Y. (2013). NF-κB signaling and bone resorption. Osteoporos. Int..

[41] Boyle W.J., Simonet W.S., Lacey D.L. (2003). Osteoclast differentiation and activation. Nat. Cell Biol..

[42] Terpos E., Christoulas D., Gavriatopoulou M. (2018). Biology and treatment of myeloma related bone disease. Metabolism.

[43] Pearse R.N., Sordillo E.M., Yaccoby S., Wong B.R., Liau D.F., Colman N., Michaeli J., Epstein J., Choi Y. (2001). Multiple myeloma disrupts the TRANCE/ osteoprotegerin cytokine axis to trigger bone destruction and promote tumor progression. Proc. Natl. Acad. Sci. USA.

[44] Farrugia A.N., Atkins G.J., To L.B., Pan B., Horvath N., Kostakis P., Findlay D.M., Bardy P., Zannettino A.C. (2003). Receptor activator of nuclear factor-kappaB ligand expression by human myeloma cells mediates osteoclast formation In Vitro and correlates with bone destruction In Vivo. Cancer Res..

[45] Spaan I., Raymakers R.A., van de Stolpe A., Peperzak V. (2018). Wnt signaling in multiple myeloma: A central player in disease with therapeutic potential. J. Hematol. Oncol..

[46] Qiang Y.-W., Chen Y., Stephens O., Brown N., Chen B., Epstein J., Barlogie B., Shaughnessy J.D. (2008). Myeloma-derived Dickkopf-1 disrupts Wnt-regulated osteoprotegerin and RANKL production by osteoblasts: A potential mechanism underlying osteolytic bone lesions in multiple myeloma. Blood.

[47] Standal T., Seidel C., Hjertner Ø., Plesner T., Sanderson R.D., Waage A., Borset M., Sundan A. (2002). Osteoprotegerin is bound, internalized, and degraded by multiple myeloma cells. Blood.

[48] Hikata T., Takaishi H., Takito J., Hakozaki A., Furukawa M., Uchikawa S., Kimura T., Okada Y., Matsumoto M., Yoshimura A. (2009). PIAS3 negatively regulates RANKL-mediated osteoclastogenesis directly in osteoclast precursors and indirectly via osteoblasts. Blood.

[49] Shay G., Tauro M., Loiodice F., Tortorella P., Sullivan D.M., Hazlehurst L.A., Lynch C.C. (2017). Selective inhibition of matrix metalloproteinase-2 in the multiple myeloma-bone microenvironment. Oncotarget.

[50] Arai A., Mizoguchi T., Harada S., Kobayashi Y., Nakamichi Y., Yasuda H., Penninger J.M., Yamada K., Udagawa N., Takahashi N. (2012). Fos plays an essential role in the upregulation of RANK expression in osteoclast precursors within the bone microenvironment. J. Cell Sci..

[51] Rossi M., Pitari M.R., Amodio N., di Martino M.T., Conforti F., Leone E., Botta C., Paolino F.M., Del Giudice T., Iuliano E. (2012). miR-29b negatively regulates human osteoclastic cell differentiation and function: Implications for the treatment of multiple myeloma-related bone disease. J. Cell. Physiol..

[52] Baron R., Kneissel M. (2013). WNT signaling in bone homeostasis and disease: From human mutations to treatments. Nat. Med..

[53] Patel S., Alam A., Pant R., Chattopadhyay S. (2019). Wnt signaling and its significance within the tumor microenvironment: Novel therapeutic insights. Front. Immunol..

[54] Bennett C.N., Longo K.A., Wright W.S., Suva L.J., Lane T.F., Hankenson K.D., MacDougald O.A. (2005). Regulation of osteoblastogenesis and bone mass by Wnt10b. Proc. Natl. Acad. Sci. USA.

[55] Almeida M., Han L., Bellido T., Manolagas S.C., Kousteni S. (2005). Wnt proteins prevent apoptosis of both uncommitted osteoblast progenitors and differentiated osteoblasts by β-catenin-dependent and -independent signaling cascades involving Src/ERK and phosphatidylinositol 3-Kinase/AKT. J. Biol. Chem..

[56] Gavriatopoulou M., Dimopoulos M.-A., Christoulas D., Migkou M., Iakovaki M., Gkotzamanidou M., Terpos E. (2009). Dickkopf-1: A suitable target for the management of myeloma bone disease. Expert Opin. Targets.

[57] Mao B., Wu W., Davidson G., Marhold J., Li M., Mechler B.M., Delius H., Hoppe D., Stannek P., Walter C. (2002). Kremen proteins are Dickkopf receptors that regulate Wnt/β-catenin signalling. Nat. Cell Biol..

[58] Gunn W.G., Conley A., Deininger L., Olson S.D., Prockop D.J., Gregory C.A. (2006). A crosstalk between myeloma cells and marrow stromal cells stimulates production of DKK1 and interleukin-6: A potential role in the development of lytic bone disease and tumor progression in multiple myeloma. Stem Cells.

[59] Colucci S., Brunetti G., Oranger A., Mori G.M., Sardone F., Specchia G., Rinaldi E., Curci P., Liso V., Passeri G. (2011). Myeloma cells suppress osteoblasts through sclerostin secretion. Blood Cancer J..

[60] Sutherland M.K., Geoghegan J.C., Yu C., Turcott E., Skonier J.E., Winkler D.G., Latham J.A. (2004). Sclerostin promotes the apoptosis of human osteoblastic cells: A novel regulation of bone formation. Bone.

[61] Winkler D.G., Sutherland M.K., Geoghegan J.C., Yu C., Hayes T., Skonier J.E., Shpektor D., Jonas M., Kovacevich B.R., Staehling-Hampton K. (2003). Osteocyte control of bone formation via sclerostin, a novel BMP antagonist. EMBO J..

[62] Brunetti G., Oranger A., Mori G., Specchia G., Rinaldi E., Curci P., Zallone A., Rizzi R., Grano M., Colucci S. (2011). Sclerostin is overexpressed by plasma cells from multiple myeloma patients. Ann. N. Y. Acad. Sci..

[63] Terpos E., Christoulas D., Katodritou E., Bratengeier C., Gkotzamanidou M., Michalis E., Delimpasi S., Pouli A., Meletis J., Kastritis E. (2012). Elevated circulating sclerostin correlates with advanced disease features and abnormal bone remodeling in symptomatic myeloma: Reduction post-bortezomib monotherapy. Int. J. Cancer.

[64] Fan F., Deng R., Qiu L., Wen Q., Zeng Y., Gao L., Zhang C., Kong P., Zhong J., Zeng N. (2019). miR-203a-3p.1 is involved in the regulation of osteogenic differentiation by directly targeting Smad9 in MM-MSCs. Oncol. Lett..

[65] Liu H., Peng F., Liu Z., Jiang F., Li L., Gao S., Wang G., Song J., Ruan E., Shao Z. (2016). CYR61/CCN1 stimulates proliferation and differentiation of osteoblasts in vitro and contributes to bone remodelling In Vivo in myeloma bone disease. Int. J. Oncol..

[66] Giuliani N., Colla S., Morandi F., Lazzaretti M., Sala R., Bonomini S., Grano M., Colucci S., Svaldi M., Rizzoli V. (2005). Myeloma cells block RUNX2/CBFA1 activity in human bone marrow osteoblast progenitors and inhibit osteoblast formation and differentiation. Blood.

[67] Trotter T.N., Li M., Pan Q., Peker D., Rowan P.D., Li J., Zhan F., Suva L.J., Javed A., Yang Y. (2015). Myeloma cell–derived Runx2 promotes myeloma progression in bone. Blood.

[68] Baniwal S.K., Shah P.K., Shi Y., Haduong J.H., Declerck Y.A., Gabet Y., Frenkel B. (2011). Runx2 promotes both osteoblastogenesis and novel osteoclastogenic signals in ST2 mesenchymal progenitor cells. Osteoporos. Int..

[69] Gowda P.S., Wildman B.J., Trotter T.N., Xu X., Hao X., Hassan M.Q., Yang Y. (2018). Runx2 Suppression by miR-342 and miR-363 inhibits multiple myeloma progression. Mol. Cancer Res..

[70] Derynck R., Budi E.H. (2019). Specificity, versatility, and control of TGF-β family signaling. Sci. Signal..

[71] Lee B., Oh Y., Jo S., Kim T.-H., Ji J.D. (2019). A dual role of TGF-β in human osteoclast differentiation mediated by Smad1 versus Smad3 signaling. Immunol. Lett..

[72] Wu M., Chen G., Li Y.-P. (2016). TGF-β and BMP signaling in osteoblast, skeletal development, and bone formation, homeostasis and disease. Bone Res..

[73] Takeuchi K., Abe M., Hiasa M., Oda A., Amou H., Kido S., Harada T., Tanaka O., Miki H., Nakamura S. (2010). TGF-β inhibition restores terminal osteoblast differentiation to suppress myeloma growth. PLoS ONE.

[74] Hill C.S. (2016). Transcriptional control by the SMADs. Cold Spring Harb. Perspect. Biol..

[75] Borton A.J., Frederick J.P., Datto M.B., Wang X.-F., Weinstein R.S. (2001). The loss of Smad3 results in a lower rate of bone formation and osteopenia through dysregulation of osteoblast differentiation and apoptosis. J. Bone Min. Res..

[76] Yang X., Chen L., Xu X., Li C., Huang C., Deng C.-X. (2001). TGF-β/Smad3 signals repress chondrocyte hypertrophic differentiation and are required for maintaining articular cartilage. J. Cell Biol..

[77] Fan F., Deng R., Lai S., Wen Q., Zeng Y., Gao L., Liu Y., Kong P., Zhong J., Su Y. (2019). Inhibition of microRNA-221-5p induces osteogenic differentiation by directly targeting smad3 in myeloma bone disease mesenchymal stem cells. Oncol. Lett..

[78] Gooding S., Olechnowicz S.W.Z., Morris E.V., Armitage A.E., Arezes J., Frost J., Repapi E., Edwards J.R., Ashley N., Waugh C. (2019). Transcriptomic profiling of the myeloma bone-lining niche reveals BMP signalling inhibition to improve bone disease. Nat. Commun..

[79] Ryoo H.-M., Lee M.-H., Kim Y.-J. (2006). Critical molecular switches involved in BMP-2-induced osteogenic differentiation of mesenchymal cells. Gene.

[80] Standal T., Abildgaard N., Fagerli U.-M., Stordal B., Hjertner Ø., Borset M., Sundan A. (2006). HGF inhibits BMP-induced osteoblastogenesis: Possible implications for the bone disease of multiple myeloma. Blood.

[81] Retting K.N., Song B., Yoon B.S., Lyons K.M. (2009). BMP canonical Smad signaling through Smad1 and Smad5 is required for endochondral bone formation. Development.

[82] Xu S., Santini G.C., de Veirman K., Broek I.V., Leleu X., de Becker A., van Camp B., Vanderkerken K., Van Riet I. (2013). Upregulation of miR-135b is involved in the impaired osteogenic differentiation of mesenchymal stem cells derived from multiple myeloma patients. PLoS ONE.

[83] Akhmetshina A., Palumbo K., Dees C., Bergmann C., Venalis P., Zerr P., Horn A., Kireva T., Beyer C., Zwerina J. (2012). Activation of canonical Wnt signalling is required for TGF-β-mediated fibrosis. Nat. Commun..

[84] Letamendia A., Labbé E., Attisano L. (2001). Transcriptional regulation by Smads: Crosstalk between the TGF-beta and Wnt pathways. J. Bone J. Surg..

[85] Wu S., He X., Li M., Shi F., Wu D., Pan M., Guo M., Zhang R., Luo S., Gu N. (2016). MiRNA-34a overexpression inhibits multiple myeloma cancer stem cell growth in mice by suppressing Tgifam. J. Transl. Res..

[86] Melhuish T.A., Gallo C.M., Wotton D. (2001). TGIF2 interacts with histone deacetylase 1 and represses transcription. J. Biol. Chem..

[87] Colombo M., Mirandola L., Platonova N., Apicella L., Basile A., Figueroa A.J., Cobos E., Chiriva-Internati M., Chiaramonte R. (2013). Notch-directed microenvironment reprogramming in myeloma: A single path to multiple outcomes. Leukemia.

[88] Xie Y., Zhang L., Gao Y., Ge W., Tang P. (2015). The multiple roles of Microrna-223 in regulating bone metabolism. Molecules.

[89] Berenstein R., Nogai A., Waechter M., Blau O., Kuehnel A., Schmidt-Hieber M., Kunitz A., Pezzutto A., Dörken B., Blau I.W. (2016). Multiple myeloma cells modify VEGF/IL-6 levels and osteogenic potential of bone marrow stromal cells via Notch/miR-223. Mol. Carcinog..

[90] Tsukamoto S., Løvendorf M.B., Park J., Salem K.Z., Reagan M.R., Manier S., Zavidij O., Rahmat M., Huynh D., Takagi S. (2018). Inhibition of microRNA-138 enhances bone formation in multiple myeloma bone marrow niche. Leukemia.

[91] Strzelecka-Kiliszek A., Mebarek S., Roszkowska M., Buchet R., Magne D., Pikula S. (2017). Functions of Rho family of small GTPases and Rho-associated coiled-coil kinases in bone cells during differentiation and mineralization. Biochim. Biophys. Acta Gen. Subj..

[92] McBeath R., Pirone D.M., Nelson C.M., Bhadriraju K., Chen C.S. (2004). Cell shape, cytoskeletal tension, and RhoA regulate stem cell lineage commitment. Dev. Cell.

[93] Gai Z., Gui T., Muragaki Y. (2011). The function of TRPS1 in the development and differentiation of bone, kidney, and hair follicles. Histol. Histopathol..

[94] Zhang Y., Xie R.-L., Gordon J.A.R., Leblanc K.T., Stein J.L., Lian J.B., Van Wijnen A.J., Stein G.S. (2012). Control of mesenchymal lineage progression by MicroRNAs targeting skeletal gene regulators Trps1 and Runxj. Biol. Chem..

[95] Zaman G., Staines K.A., Farquharson C., Newton P.T., Dudhia J., Chenu C., Pitsillides A.A., Dhoot G.K. (2015). Expression of Sulf1 and Sulf2 in cartilage, bone and endochondral fracture healing. Histochem. Cell Biol..

[96] Van Niel G., D’Angelo G., Raposo G. (2018). Shedding light on the cell biology of extracellular vesicles. Nat. Rev. Mol. Cell Biol..

[97] Moloudizargari M., Abdollahi M., Asghari M.H., Zimta A.A., Neagoe I.B., Nabavi S.M. (2019). The emerging role of exosomes in multiple myeloma. Blood Rev..

[98] Colombo M., Giannandrea D., Lesma E., Basile A., Chiaramonte R. (2019). Extracellular vesicles enhance multiple myeloma metastatic dissemination. Int. J. Mol. Sci..

[99] Liu Y., Zhu X.-J., Zeng C., Wu P.-H., Wang H.-X., Chen Z.-C., Li Q.-B. (2013). Microvesicles secreted from human multiple myeloma cells promote angiogenesis. Acta Pharm. Sin..

[100] Wang J., de Veirman K., de Beule N., Maes K., de Bruyne E., van Valckenborgh E., Vanderkerken K., Menu E. (2015). The bone marrow microenvironment enhances multiple myeloma progression by exosome-mediated activation of myeloid-derived suppressor cells. Oncotarget.

[101] Raimondi L., De Luca A., Amodio N., Manno M., Raccosta S., Taverna S., Bellavia D., Naselli F., Fontana S., Schillaci O. (2015). Involvement of multiple myeloma cell-derived exosomes in osteoclast differentiation. Oncotarget.

[102] Raimondo S., Saieva L., Vicario E., Pucci M., Toscani D., Manno M., Raccosta S., Giuliani N., Alessandro R., Raccosta S. (2019). Multiple myeloma-derived exosomes are enriched of amphiregulin (AREG) and activate the epidermal growth factor pathway in the bone microenvironment leading to osteoclastogenesis. J. Hematol. Oncol..

[103] Faict S., Muller J., De Veirman K., De Bruyne E., Maes K., Vrancken L., Heusschen R., De Raeve H., Schots R., Vanderkerken K. (2018). Exosomes play a role in multiple myeloma bone disease and tumor development by targeting osteoclasts and osteoblasts. Blood Cancer J..

[104] Liu Z., Liu H., Li Y., Shao Q., Chen J., Song J., Fu R. (2019). Multiple myeloma-derived exosomes inhibit osteoblastic differentiation and improve IL-6 secretion of BMSCs from multiple myeloma. J. Investig. Med..

[105] Zhang L., Lei Q., Wang H., Xu C., Liu T., Kong F., Yang C., Yan G., Sun L., Zhao A. (2019). Tumor-derived extracellular vesicles inhibit osteogenesis and exacerbate myeloma bone disease. Theranostics.

[106] Nolte’t Hoen E.N., Buermans H.P., Waasdorp M., Stoorvogel W., Wauben M.H., Hoen P.A. (2012). Deep sequencing of RNA from immune cell-derived vesicles uncovers the selective incorporation of small non-coding RNA biotypes with potential regulatory functions. Nucleic Acids Res..

[107] Valadi H., Ekström K., Bossios A., Sjöstrand M., Lee J.J., Tvall J.O. (2007). Exosome-mediated transfer of mRNAs and microRNAs is a novel mechanism of genetic exchange between cells. Nat. Cell Biol..

[108] Santangelo L., Giurato G., Cicchini C., Montaldo C., Mancone C., Tarallo R., Battistelli C., Alonzi T., Weisz A., Tripodi M. (2016). The RNA-binding protein SYNCRIP is a component of the hepatocyte exosomal machinery controlling MicroRNA sorting. Cell Rep..

[109] Frassanito M.A., DeSantis V., Di Marzo L., Craparotta I., Beltrame L., Marchini S., Annese T., Visino F., Arciuli M., Saltarella I. (2019). Bone marrow fibroblasts overexpress miR-27b and miR-214 in step with multiple myeloma progression, dependent on tumour cell-derived exosomes. J. Pathol..

[110] Zhang Z.-Y., Li Y.-C., Geng C.-Y., Wang H.-J., Chen W.-M. (2019). Potential relationship between clinical significance and serum exosomal miRNAs in patients with multiple myeloma. Biomed. Res. Int..

[111] Raimondo S., Urzì O., Conigliaro A., Bosco G.L., Parisi S., Carlisi M., Siragusa S., Raimondi L., De Luca A., Giavaresi G. (2020). Extracellular vesicle microRNAs contribute to the osteogenic inhibition of mesenchymal stem cells in multiple myeloma. Cancers.

[112] Zhang Z., Li Y., Geng C., Zhou H., Gao W., Chen W. (2019). Serum exosomal microRNAs as novel biomarkers for multiple myeloma. Hematol. Oncol..

[113] Li S., Wu X., Pei Y., Wang W., Zheng K., Qiu E., Zhang X. (2019). PTHR1May be involved in progression of osteosarcoma by regulating miR-124–3p-AR-Tgfb1i1, miR-27a-3p-PPARG-Abca1, and miR-103/590–3p-AXIN2Axes. DNA Cell Biol..

[114] Karousi P., Adamopoulos P.G., Papageorgiou S.G., Pappa V., Scorilas A., Kontos C.K. (2020). A novel, mitochondrial, internal tRNA-derived RNA fragment possesses clinical utility as a molecular prognostic biomarker in chronic lymphocytic leukemia. Clin. Biochem..

[115] Karousi P., Katsaraki K., Papageorgiou S.G., Pappa V., Scorilas A., Kontos C.K. (2019). Identification of a novel tRNA-derived RNA fragment exhibiting high prognostic potential in chronic lymphocytic leukemia. Hematol. Oncol..

[116] Katsaraki K., Artemaki P.I., Papageorgiou S.G., Pappa V., Scorilas A., Kontos C.K. (2019). Identification of a novel, internal tRNA-derived RNA fragment as a new prognostic and screening biomarker in chronic lymphocytic leukemia, using an innovative quantitative real-time PCR assay. Leuk. Res..

[117] Kontos C.K., Vasilatou D., Papageorgiou S.G., Scorilas A. (2006). Translation Regulation by microRNAs in Acute Leukemia. Encyclopedia of Molecular Cell Biology and Molecular Medicine.

[118] Hao M., Zang M., Zhao L., Deng S., Xu Y., Qi F., An G., Qin Y., Sui W., Li F. (2016). Serum high expression of miR-214 and miR-135b as novel predictor for myeloma bone disease development and prognosis. Oncotarget.

[119] Yuan R., Liu N., Yang J., Peng J., Liu L., Guo X. (2018). The expression and role of miR-181a in multiple myeloma. Medicine.

[120] Yan X., Gao M., Zhang P., Ouyang G., Mu Q., Xu K. (2020). MiR-181a functions as an oncogene by regulating CCND1 in multiple myeloma. Oncol. Lett..

[121] Van Andel H., Kocemba K.A., Spaargaren M., Pals S.T. (2019). Aberrant Wnt signaling in multiple myeloma: Molecular mechanisms and targeting options. Leukemia.

[122] Gavriatopoulou M., Terpos E., Ntanasis-Stathopoulos I., Malandrakis P., Eleutherakis-Papaiakovou E., Papatheodorou A., Kanellias N., Migkou M., Fotiou D., Dialoupi I. (2020). Consolidation with carfilzomib, lenalidomide, and dexamethasone (KRd) following ASCT results in high rates of minimal residual disease negativity and improves bone metabolism, in the absence of bisphosphonates, among newly diagnosed patients with multiple myeloma. Blood Cancer J..

[123] Terpos E., Katodritou E., Symeonidis A., Zagouri F., Gerofotis A., Christopoulou G., Gavriatopoulou M., Christoulas D., Ntanasis-Stathopoulos I., Kourakli A. (2019). Effect of induction therapy with lenalidomide, doxorubicin and dexamethasone on bone remodeling and angiogenesis in newly diagnosed multiple myeloma. Int. J. Cancer.

[124] Terpos E., Kastritis E., Ntanasis-Stathopoulos I., Christoulas D., Papatheodorou A., Eleutherakis-Papaiakovou E., Kanellias N., Fotiou D., Ziogas D.C., Migkou M. (2019). Consolidation therapy with the combination of bortezomib and lenalidomide (VR) without dexamethasone in multiple myeloma patients after transplant: Effects on survival and bone outcomes in the absence of bisphosphonates. Am. J. Hematol..

[125] Terpos E., Christoulas D., Kastritis E., Roussou M., Migkou M., Eleutherakis-Papaiakovou E., Gavriatopoulou M., Gkotzamanidou M., Kanellias N., Manios E. (2013). VTD consolidation, without bisphosphonates, reduces bone resorption and is associated with a very low incidence of skeletal-related events in myeloma patients post ASCT. Leukemia.

[126] Kleber M., Ntanasis-Stathopoulos I., Dimopoulos M.A., Terpos E. (2019). Monoclonal antibodies against RANKL and sclerostin for myeloma-related bone disease: Can they change the standard of care?. Expert Rev. Hematol..

[127] Loredana S., Santo L., Wein M.N., Hu D.Z., Cirstea D.D., Nemani N., Tai Y.-T., Raines S.E., Kuhstoss S.A., Munshi N.C. (2016). Regulation of sclerostin expression in multiple myeloma by Dkk-1: A potential therapeutic strategy for myeloma bone disease. J. Bone Min. Res..

[128] McDonald M.M., Reagan M.R., Youlten S.E., Mohanty S.T., Seckinger A., Terry R.L., Pettitt J.A., Simic M.K., Cheng T.L., Morse A. (2017). Inhibiting the osteocyte-specific protein sclerostin increases bone mass and fracture resistance in multiple myeloma. Blood.

[129] Iyer S.P., Beck J.T., Stewart A.K., Shah J., Kelly K.R., Isaacs R., Bilic S., Sen S., Munshi N.C. (2014). A Phase IB multicentre dose-determination study of BHQ 880 in combination with anti-myeloma therapy and zoledronic acid in patients with relapsed or refractory multiple myeloma and prior skeletal-related events. Br. J. Haematol..

[130] Karousi P., Artemaki P.I., Sotiropoulou C.D., Christodoulou S., Scorilas A., Kontos C.K. (2020). Identification of two novel circular RNAs deriving from BCL2L12 and investigation of their potential value as a molecular signature in colorectal cancer. Int. J. Mol. Sci..

[131] Fortunato O., Iorio M.V. (2020). The therapeutic potential of MicroRNAs in cancer: Illusion or opportunity?. Pharmaceuticals.

[132] Hong D.S., Kang Y.-K., Borad M., Sachdev J., Ejadi S., Lim H.Y., Brenner A.J., Park K., Lee J.-L., Kim T.-Y. (2020). Phase 1 study of MRX34, a liposomal miR-34a mimic, in patients with advanced solid tumours. Br. J. Cancer.

